# Automatic Branch–Leaf Segmentation and Leaf Phenotypic Parameter Estimation of Pear Trees Based on Three-Dimensional Point Clouds

**DOI:** 10.3390/s23094572

**Published:** 2023-05-08

**Authors:** Haitao Li, Gengchen Wu, Shutian Tao, Hao Yin, Kaijie Qi, Shaoling Zhang, Wei Guo, Seishi Ninomiya, Yue Mu

**Affiliations:** 1 Academy for Advanced Interdisciplinary Studies, Collaborative Innovation Center for Modern Crop Production Co-Sponsored by Province and Ministry, Nanjing Agricultural University, Nanjing 210095, China; 2 College of Artificial Intelligence, Nanjing Agricultural University, Nanjing 210095, China; 3 Centre of Pear Engineering Technology Research, State Key Laboratory of Crop Genetics & Germplasm Enhancement and Utilization, Nanjing Agricultural University, Nanjing 210095, China; 4 Graduate School of Agricultural and Life Sciences, The University of Tokyo, 1-1-1 Midori-cho, Tokyo 188-0002, Japan

**Keywords:** pear canopy, point cloud segmentation, leaf phenotype

## Abstract

The leaf phenotypic traits of plants have a significant impact on the efficiency of canopy photosynthesis. However, traditional methods such as destructive sampling will hinder the continuous monitoring of plant growth, while manual measurements in the field are both time-consuming and laborious. Nondestructive and accurate measurements of leaf phenotypic parameters can be achieved through the use of 3D canopy models and object segmentation techniques. This paper proposed an automatic branch–leaf segmentation pipeline based on lidar point cloud and conducted the automatic measurement of leaf inclination angle, length, width, and area, using pear canopy as an example. Firstly, a three-dimensional model using a lidar point cloud was established using SCENE software. Next, 305 pear tree branches were manually divided into branch points and leaf points, and 45 branch samples were selected as test data. Leaf points were further marked as 572 leaf instances on these test data. The PointNet++ model was used, with 260 point clouds as training input to carry out semantic segmentation of branches and leaves. Using the leaf point clouds in the test dataset as input, a single leaf instance was extracted by means of a mean shift clustering algorithm. Finally, based on the single leaf point cloud, the leaf inclination angle was calculated by plane fitting, while the leaf length, width, and area were calculated by midrib fitting and triangulation. The semantic segmentation model was tested on 45 branches, with a mean *Precision*_sem_, mean *Recall*_sem_, mean *F*1*-score*, and mean Intersection over Union (*IoU*) of branches and leaves of 0.93, 0.94, 0.93, and 0.88, respectively. For single leaf extraction, the *Precision*_ins_, *Recall*_ins_, and mean coverage (*mCoV*) were 0.89, 0.92, and 0.87, respectively. Using the proposed method, the estimated leaf inclination, length, width, and area of pear leaves showed a high correlation with manual measurements, with correlation coefficients of 0.94 (root mean squared error: 4.44°), 0.94 (root mean squared error: 0.43 cm), 0.91 (root mean squared error: 0.39 cm), and 0.93 (root mean squared error: 5.21 cm^2^), respectively. These results demonstrate that the method can automatically and accurately measure the phenotypic parameters of pear leaves. This has great significance for monitoring pear tree growth, simulating canopy photosynthesis, and optimizing orchard management.

## 1. Introduction

Leaves are the primary organs for photosynthesis and respiration in plants, especially fruit trees, and account for the largest proportion in the tree crown. They play a key role in the growth and development of plants, making their characteristics important for characterizing plant growth conditions [1]. Thus, automatic extraction of phenotypic parameters from leaves is essential for monitoring fruit tree growth [2].

Traditional methods for extracting leaf parameters often require manual measurement, which are time-consuming, laborious, and destructive [3]. Devices based on radiation transmittance measurement have been used for canopy porosity studies, but the sensors could only been put at sample points or line transect, which still takes a lot of sampling time to analyze the complete 3D canopy [4]. Although image-based methods are low-cost and fast, the extraction of leaf phenotypes may be limited in complex scenarios with severe canopy overlap [5,6]. Fortunately, the use of ToF (Time of Flight) camera [7] and lidar in agriculture and forestry allows for the quick and accurate acquisition of three-dimensional information about the canopy, making the extraction of leaf phenotypic parameters as well as plants’ (trees’) volume efficient and accurate [8,9,10,11]. However, extracting leaves from canopy point cloud models to realize single leaf measurement is challenging [12]. The common strategy is to separate branches and leaves first, then extract single leaves, and finally measure leaf phenotypic parameters.

In recent years, branch–leaf segmentation based on point clouds are continuously developing, from using the difference in laser reflection intensity based on branches and leaves, to the use of difference in their spatial structure characteristics, and further obtaining more features through deep learning methods. Cote et al. [13] attempted to classify evergreen conifer trunks and leaves using their different infrared spectral responses. However, the separation of branches and leaves by use of single intensity information is empirical. Xu et al. [14] calculated the shortest path from each point to the root point of the tree, and then used a threshold to distinguish the point cloud of the branches and the leaves. However, the accuracy of this segmentation method is not high enough. Su et al. [15] proposed an extraction algorithm that combines classification and segmentation based on K-means clustering and random sample consensus algorithm (RANSAC) to divide canopy point clouds into branches and leaves. Tang et al. [16] separated the branches and leaves by using both the similarity of principal direction between neighboring points and distribution density of points. Hu et al. [17] used the SegNet network to separate branches and leaves in the depth images and then extract leaf point clouds. Compared with using the discrepancy between reflection intensity and simple spatial structure characteristics of branch and leaf, it is more accurate to segment them through a deep learning model.

In terms of single leaf segmentation based on point clouds, it has been conducted in various plants to trees. For the leaf segmentation of plants, Xia et al. [18] used mean-shift clustering to segment objects from the background and active contour models, while calculating gradient vector field to segment leaves in situ in a greenhouse. Li et al. [19] proposed a single leaf segmentation method based on three-dimensional filtering and facet region growth, which can better segment overlapping leaves. In the leaf segmentation of crops, Duan et al. [20] used the octree algorithm to segment wheat point clouds by single leaf, and estimated phenotypic parameters. Jin et al. [21] proposed the median normalized vector growth algorithm to segment maize stems and leaves based on lidar point clouds through four steps: pre-treatment, stem growth, leaf growth, and post-treatment. In the leaf segmentation of (fruit) trees, Liu et al. [22] used the dynamic K threshold to segment single leaves on the branches of apple trees. However, the point cloud of the branches was not removed before single leaf segmentation, leaving some noise in the point clouds of leaves. Similarly, Wu et al. [23] acquired the point clouds of apple and orange canopies, and adopted Affinity Propagation algorithm to realize the separation of leaves. Koma et al. [24] extracted leaves by region growing for the lidar point clouds of a tulip tree. In addition, the methodology of segmenting poplar single leaves in the literature [17] was also the point cloud clustering method, based on k–d tree. According to the above, we found that methods based on region growing are the most common methods for single leaf segmentation of plants and crops. However, clustering methods based on Euclidean distance are more commonly used in single leaf segmentation of (fruit) trees than plants and crops, which may be due to the larger canopy, relatively smaller leaves, and scattered leaf distribution. 

In addition to the above step-by-step extraction strategy, the application of deep learning methods makes it possible to simultaneously conduct branch–leaf semantic segmentation and segmentation of single leaf instances. Jin et al. [12] proposed a voxel-based convolutional neural network (VCNN) for the stem–leaf semantic segmentation and instance segmentation of maize from terrestrial lidar data. In a similar deep learning approach, Li et al. [25] proposed the PSegNet neural network to segment plant point clouds and trained multi-period tobacco, tomato, and sorghum point clouds using the Voxelized Farthest Point Sampling (VFPS) strategy. Li et al. [26] developed DeepSeg3Dmaize, a plant point cloud segmentation technique that integrates high-throughput data acquisition and deep learning, using PointNet to implement stem–leaf and organ instance segmentation. Compared with the previous two plant/crop models, PointNet is more widely applicable.

Based on single leaf point cloud, phenotypic parameters such as leaf length and width, perimeter, area, leaf inclination, and azimuth angle can be easily estimated. Leaf length and width are the most commonly measured parameters, which can be estimated using the distance between two points on the tip and base [27], or through convex hull [24] and bounding box methods [28,29]. However, due to leaf curvature, these methods may lead to large errors. Using the midrib of the leaf to simplify the point cloud can improve the accuracy [20]. For leaf inclination angle, it can be estimated by the angle between the ventral normal of the leaf and the zenith axis, and the least squares (LS) method are widely used for plane (line) fitting to obtain the normal (directional) vector [26,30]. As for leaf area, it can be estimated with point cloud triangulation and surface reconstruction [19,31,32].

Compared with crops and plants, due to the interlacing branches in the canopy, it is difficult to obtain high-quality canopy point cloud models of fruit trees. In addition, the leaf segmentation methods using deep learning largely rely on datasets, and the annotation of branch/leaf point cloud models of fruit trees is also challenging. Therefore, there are few studies on organ scale (branch and leaf) segmentation for fruit trees based on deep learning, much less based on leaf phenotypic characteristics’ measurement, at present. In this study, we aim to develop an automatic pipeline for single leaf extraction and measurement of fruit trees, not only efficient in point cloud processing for fruit tree canopy, but also accurate in measuring leaf phenotypic parameters. 

## 2. Materials and Methods

**Figure 1:**
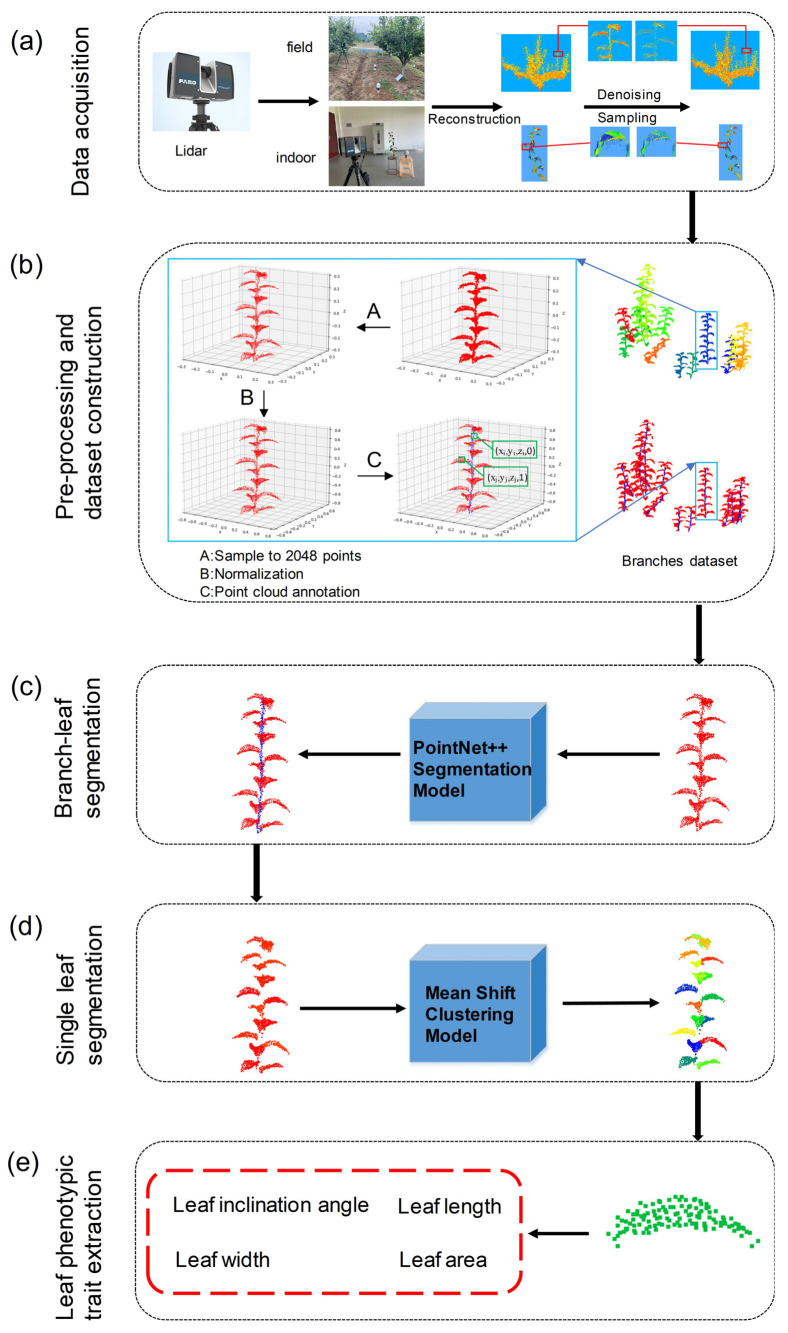
Workflow of this study: (**a**) Data acquisition; (**b**) Data pre-processing and branch level dataset construction; (**c**) Branch–leaf segmentation with PointNet++ segmentation model; (**d**) Single leaf segmentation with Mean Shift Clustering Model; (**e**) Leaf phenotypic trait extraction.

This section is comprised of five main parts: point cloud data acquisition and preprocessing, construction of the branch level point cloud dataset, branch and leaf point cloud segmentation using deep learning, leaf point cloud segmentation using clustering, and estimation of leaf phenotypic parameters (as shown in Figure 1). The related algorithms and samples are available at https://github.com/haitao971028/branch-leaf_segmentation_and_leaf_traits_extraction (accessed on 1 March 2021). 

### 2.1. Data Acquisition

Pear trees (*Pyrus pyrifolia* ‘Cuiguan’) with “Y” shape and 7 years of age were taken as materials, planted in Baima Scientific Research Base of Nanjing Agricultural University, Lishui District, Nanjing, Jiangsu Province, China. 

In order to obtain accurate point cloud data of pear trees, the three-dimensional laser scanner FARO Focus^3D^ S70 (FARO Technologies, Inc., Lake Mary, FL, USA) was used for point cloud data acquisition. The scanning was conducted in late June 2022 under clear weather conditions, with no wind or light breeze. Due to the severe obscuration between the leaves and branches of the pear tree, the scanner was placed on a tripod with a height of approximately 1.6 m for multi-site scanning, in order to obtain all-round point cloud data. The field of view was 360° horizontal × 300° vertical, and the scanning distance was approximately 5 m (as shown in Table 1). The multi-site cloud registration was completed using FARO SCENE software (FARO Technologies, Inc., Lake Mary, FL, USA), and a total of 20 pear trees were scanned. To conduct subsequent quantitative evaluation on the estimation of phenotypic parameters, 5 branch samples were randomly selected from the pear trees for indoor scanning (as shown in Figure 1a), with the scanning parameters set the same as those in the field. Additionally, a total of 50 leaves were selected from the 5 branch samples, and the corresponding leaf dip angle, length, width, and area were manually measured as the true values.

### 2.2. Dataset Construction

#### 2.2.1. Data Preprocessing

Using the Cloud Compare V2 (CC) point cloud visualization software (http://www.cloudcompare.org/, accessed on 1 March 2021) and the Point Cloud Library (PCL, Kitware Inc., Tallahassee, FL, USA), the denoising and thinning of the pear tree point clouds were completed [33]. The specific processes were as follows: Firstly, the ground and trunk point clouds were manually removed using CC to obtain the whole canopy point clouds. Then, Statistical Outlier Removal algorithm in the PCL was used to remove outliers. Finally, the point cloud was voxelized to implement thinning, with the length, width, and height of the voxels set to 0.003 m.

We used a semi-automated method to extract branches from the canopy. Firstly, CC software was used to select some relatively complete clusters of branches from the whole canopy for preliminary extraction. Then, cluster segmentation of branches was performed based on Density-Based Spatial Clustering of Applications with Noise (DBSCAN) [34] and K-Nearest Neighbor (KNN) [35] algorithm to obtain single branches. For a few serious overlapping branches, we manually segmented them by CC software. A total of 373 branch samples, including 5 samples scanned indoors, were taken from the 20 pear canopies point cloud models to establish the branch dataset. The number of points for each branch ranged from 3 k to 10 k. To improve model training efficiency and retain the structural characteristics of branches, the Farthest Point Sampling (FPS) method was used to sample the branch point cloud to 2048 points. The point cloud was then normalized with the origin as the center into a cube with a side length of 2 m. To avoid affecting subsequent parameter measurement, the conversion parameter from the original point cloud model to the normalized output was recorded, and was later used to reverse the normalized point cloud to original scale and measure its real size.

#### 2.2.2. Point Cloud Labeling

After data preprocessing, point cloud annotation is necessary to implement the following model training. In this study, labels were set at the point level. Since the data samples consisted only of branches and leaves, we assigned a label of “1” to point clouds belonging to leaves and “0” to branch point clouds (as shown in Figure 1b). The labeling was done using CC software.

The format of point cloud data in this study is an *n* × 4 matrix, where n is the number of points in the sample. The matrix consists of four columns: the x, y, and z coordinates of the points, and a label column with values of 0 or 1.

#### 2.2.3. Dataset Partitioning

In order to ensure the quality of the dataset, 373 branches were selected by considering the diversity of leaf density, leaf distribution, and the completeness of the branch and leaves. Finally, we obtained a total of 305 high-quality branch samples (including 5 indoor samples). Additionally, we conducted statistics on this dataset. The length of all branches ranged from 0.25 m to 1.13 m, and the number of leaves on each branch ranged from 8 to 26, as shown in Table 2. Out of the 305 samples, 260 samples (all infield) were randomly selected for training, and another 45 (40 infield and 5 indoor) were used as test samples.

### 2.3. Branch–Leaf Segmentation Based on PointNet++ Model

#### 2.3.1. PointNet++ Segmentation Model

PointNet++ [36] is a deep neural network capable of directly processing disordered point cloud data. It is an upgraded version of PointNet [37] that addresses the limitations of the PointNet network with regard to local feature extraction. The network is primarily used for point cloud classification and segmentation. In this experiment, the segmentation network of PointNet++ is utilized for branch–leaf segmentation.

**Figure 2:**
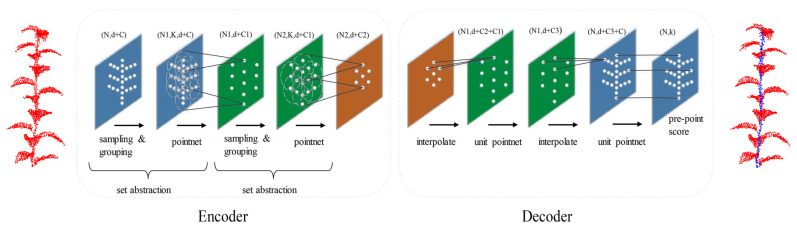
Structure of PointNet++ segmentation network. N represents the number of points, K represents the number of groups, d represents the coordinate dimension, and C represents the feature dimension.

The segmentation network is comprised of an encoder and a decoder. The encoder is primarily responsible for the point cloud downsampling process, and extracting the local features of the point cloud by setting up multiple Set Abstraction structures. The Set Abstraction is composed of sampling, grouping, and PointNet modules, which eventually output a point cloud with global features. The decoder, on the other hand, is responsible for the upsampling process. The downsampled points are restored through distance-based interpolation, and the characteristics of each point are calculated based on the KNN, which are then sent to Softmax to achieve point-level classification. Figure 2 shows the structure of the segmentation network.

#### 2.3.2. Model Training

The PointNet++ model was trained using the PyTorch (https://pytorch.org/) framework. The training set was input the network with a batch size of 4. The initial learning rate was set to 0.001 and dynamically adjusted using the ADAM optimizer and stochastic gradient descent (SGD). The momentum was set to 0.9, and the weight attenuation coefficient was set to 0.001.

In this experiment, the PointNet++ segmentation network was iterated for 500 epochs. The network was trained on an Ubuntu 16.04 OS, with an Intel Xeon E5-2698V4 CPU, 256 GB of memory, and NVIDIA Tesla V100 GPU.

### 2.4. Single Leaf Segmentation Based on Mean Shift Clustering

PointNet++ performed semantic segmentation of the points of branches and leaves at branch level, but did not segment single leaves. Therefore, we then utilized the coordinate information of the point cloud to conduct mean shift clustering [38] in three-dimensional space for instance segmentation of single leaves. Unlike other clustering algorithms, the mean shift algorithm is based on centroids. It can identify the dense center of data points by radius and cluster according to the density center without specifying a number of clusters [39].

The algorithm requires setting the key parameter radius. To ensure that the clustering center is closer to the leaf centroid, the size of the radius was set as the radius of the circumscribed sphere of the leaf point cloud. In this study, three different radius sizes of 35 mm, 45 mm, and 55 mm were set, based on the actual size of pear tree leaves.

### 2.5. Estimation of Phenotypic Parameters

**Figure 3:**
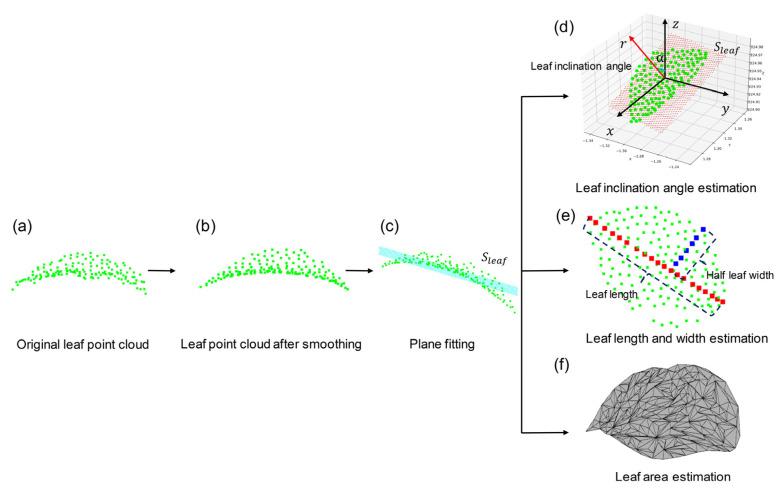
Schematic diagram of phenotypic parameters estimation based on single leaf point cloud. (**a**) Original leaf point cloud; (**b**) Leaf point cloud after smoothing using MLS; (**c**) Leaf point cloud plane fitting; (**d**) Leaf inclination angle estimation; (**e**) Leaf length and width estimation; (**f**) Leaf area estimation. *S_leaf_* in (**c**,**d**) is the leaf point cloud fitting plane.

Based on the single leaf point cloud, four phenotypic parameters, including leaf inclination angle, leaf length, leaf width, and leaf area, were estimated, as shown in Figure 1e and Figure 3. When using multi-station lidar scan for registering, the leaf surface in point cloud may not be smooth due to wind and registration errors, which has a significant impact on leaf surface reconstruction in the later stage. Therefore, the Moving Least Squares (MLS) method [40] was employed to resample the point clouds (i.e., smoothing) before parameter estimation, as shown in Figure 3b.

#### 2.5.1. Estimation of Leaf Inclination Angle

The leaf inclination angle is the angle between the ventral normal *γ* of the leaf and the zenith axis z, ranging from 0° to 90°. In this study, the normal vector *r* of leaf fitting plane *S_leaf_* was used to approximate the normal vector of the leaf point cloud, and the leaf inclination α was the angle between *r* and *z*, as shown in Figure 3d. To obtain *S_leaf_* and *r*, the least squares method was used [30].

#### 2.5.2. Estimation of Leaf Length and Width

**Figure 4:**
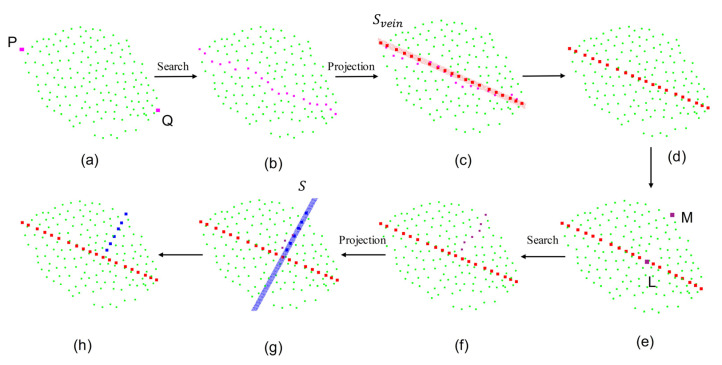
Schematic diagram of estimating leaf length and width based on midrib fitting. (**a**) The leaf base point P and the leaf tip point Q; (**b**) Approximate point cloud (purple points) of midrib; (**c**) The approximate midrib point cloud is projected onto the plane *S_vein_*; (**d**) Midrib fitting point cloud (red points) after projection; (**e**) The starting point M and the ending point L when estimating the leaf width; (**f**) Approximate point cloud (dark purple points) of estimating leaf width; (**g**) The approximate point cloud of estimating leaf width is projected onto the plane *S*; (**h**) Fitting point cloud for estimating leaf length (red points) and leaf width (blue points). *S_vein_* in (**c**) is the projection plane of midrib point cloud, and *S* in (**g**) is the widest cross section of the leaf.

In order to improve the accuracy of parameter estimation, this study proposed the midrib fitting algorithm to extract leaf length and width from point cloud, as shown in Figure 4.

Based on the morphology of pear tree leaves and the leaf point cloud, it can be estimated that the two points with the furthest distance from the leaf point cloud are the leaf base point P and the leaf tip point Q (Figure 4a). Using these two points, the K-nearest neighbor algorithm was employed to approximate the midrib. The algorithm involves the following steps:

1. Set the two points obtained above as the starting and ending points, respectively (no need to specify which is the starting point);
2. Take the starting point as the leaf base point and add it to the base point set;
3. Establish a k–d tree [41] of the leaf point cloud and search for the 1st to Kth nearest neighbor points of the base point;
4. Calculate the distance 1 between one of these neighbor points and the base point, and the distance 2 between it and the ending point. Sum up distance 1 and distance 2 and denote this as D. Repeat this until the D of all the points is calculated;
5. Find the point N that minimizes D;
6. Add N to the base point set, set N as the new base point, and remove N from the leaf point cloud;
7. Repeat steps 3, 4, 5, 6 until the base point equals the endpoint;
8. Collect the base points.

The pseudo code of the midrib fitting algorithm is as follows (Algorithm 1):

**Algorithm 1** Midrib fitting algorithm**Inputs**: Point cloud *I***Parameters**: Starting point *s*, endpoint *e*, base point *b* = *s*, and *k*.**Outputs**: Point cloud *O* after midrib fitting Define three local variables *N*, *D* and *d*

O←∅, N←(0,0,0), D←0, and d←+∞

*O.push_back* (*b*).**while** *b* ≠ *e* **do**  Establish the k–d tree of *O*.  Initialize *b’*s *k* nearest neighbors $n_{k}$  **for** each *n*, in $n_{k}$
**do**    Compute *D*, the sum of the distance between *n* and *b*, and the distance between *n* and *e*.    **if**
*D* < *d*
**then**       *N*
←
*n*       *d*
←
*D*    **end if**
  **end for**  *O*.*push_back* (*N*), and *I.erase* (*N*).  *b*
←
*N*
**end while**


After the above steps, a point cloud approximation of the midrib can be obtained (Figure 4b). To improve the fitting of the midrib, the above point cloud is projected onto the plane *S_vein_*, which passes through the points P and Q and is perpendicular to *S_leaf_*, to obtain a new point cloud of the midrib (Figure 4d). The distance between adjacent points is calculated, and the leaf length can be approximated by adding them up.

The calculation method for leaf width is similar to that of leaf length. First, the starting point M is obtained, which is a boundary point at the widest cross section of the leaf, by finding the farthest distance from the leaf point cloud to *S_vein_* (Figure 4e). Then, the distance between point M and each point in the fitted midrib point cloud is calculated. If the distance between points L and M is the shortest, L is the desired end point (Figure 4e). Finally, based on L and M, the midrib fitting algorithm can be used to acquire the point for calculating leaf width (Figure 4f). In this case, the projection plane is S (Figure 4g), which passes through point M and is perpendicular to *S_leaf_* and *S_vein_*. The distance between adjacent points is calculated, and half of the leaf width can be approximated by summing these distances.

#### 2.5.3. Estimation of Leaf Area

Greedy Projection Triangulation algorithm [32] is adopted to build the mesh of single leaf point cloud, as shown in Figure 3f.

Using Helen’s formula to calculate the area of each triangle and sum up to approximate the leaf area ${Area}_{leaf}$, the formula is as follows:

(1) $${Area}_{leaf}=\sum_{i=1}^{n}\sqrt{p_{i}(p_{i}-a_{i})(p_{i}-b_{i})(p_{i}-c_{i})}$$

(2) $$p_{i}=\frac{1}{2}\left(a_{i}+b_{i}+c_{i}\right)$$

where $a_{i},b_{i},c_{i}$ and $p_{i}$ are the three sides and half of the perimeter of the *i*th triangle, respectively.

### 2.6. Evaluation Metric

In this study, we employed various evaluation methods for the branch–leaf and single leaf segmentation. To evaluate the performance of the semantic segmentation of branches and leaves, we introduced four metrics: *Precision*_sem_, *Recall*_sem_, *F*1*-score*, and Intersection over Union (*IoU*) [42]. For each semantic class, the *IoU* reflects the degree of overlapping between the predicted results of each semantic category and the corresponding real results. *Precision*_sem_ reflects the proportion of points that the network correctly predicted in the total number of points predicted in the corresponding category. *Recall*_sem_ refers to the ratio of the number of points the network correctly predicted to the total number of points in this category. *F*1-*score* is the harmonic mean of *Precision*_sem_ and *Recall*_sem_, and its value ranges between 0 and 1. Higher values for these four indicators indicate better segmentation performance. The four metrics are defined as follows:

(3) $${Precision}_{sem}=\frac{TP}{TP+FN}$$

(4) $${Recall}_{sem}=\frac{TP}{TP+FP}$$

(5) $$F1–score=2\times \frac{{Precision}_{sem}\times {Recall}_{sem}}{{Precision}_{sem}+{Recall}_{sem}}$$

(6) $$IoU=\frac{TP}{TP+FP+FN}$$

Among them, *TP* refers to a point that is correctly predicted in this class, i.e., belongs to the same class as manually labeled. *FN* refers to a point manually labeled in this class but is incorrectly predicted to be in another class. *FP* refers to a point that is not manually labeled in this class but is predicted to belong to it. These metrics were calculated for both branches and leaves, and the averages were used for comprehensive evaluation. 

For the evaluation of single leaf segmentation, the mean coverage (*mCov*) was used [43]. *mCov* represents the average point-level *IoU* matching between predicted and manually marked instance, which is defined as follows:

(7) $$mCov(I,P)=\frac{1}{I}\sum_{m=1}^{I}\underset{n}{\max}\left(IoU(I_{m},P_{n})\right)$$

where *I* represents the number of all instances, *I_m_* represents the real point set of the *m*th instance, and *P_n_* represents the predicted point set of the *n*th instance. The calculation of *IoU* is the same as that in semantic segmentation. 

In addition to point-level evaluation, all instances with *IoU* higher than 0.5 were counted and evaluated at the instance level using two metrics: *Precision*_ins_ and *Recall*_ins_. The definitions are as follows:

(8) $${Precision}_{ins}=\frac{T}{P}$$

(9) $${Recall}_{ins}=\frac{T}{G}$$

where *T* is the number of *IoU*s greater than 0.5 in predicted instances and manually marked instances, *P* is the total number of predicted instances, and *G* is the number of manually marked instances.

The measurements of each phenotypic parameter were evaluated by correlation analysis. The error and accuracy of each parameter were quantified by calculating Root Mean Square Error (*RMSE*) and determination coefficient *R*^2^ between the estimated values and the manually measured values.

## 3. Results

### 3.1. Branch–Leaf Segmentation

**Figure 5:**
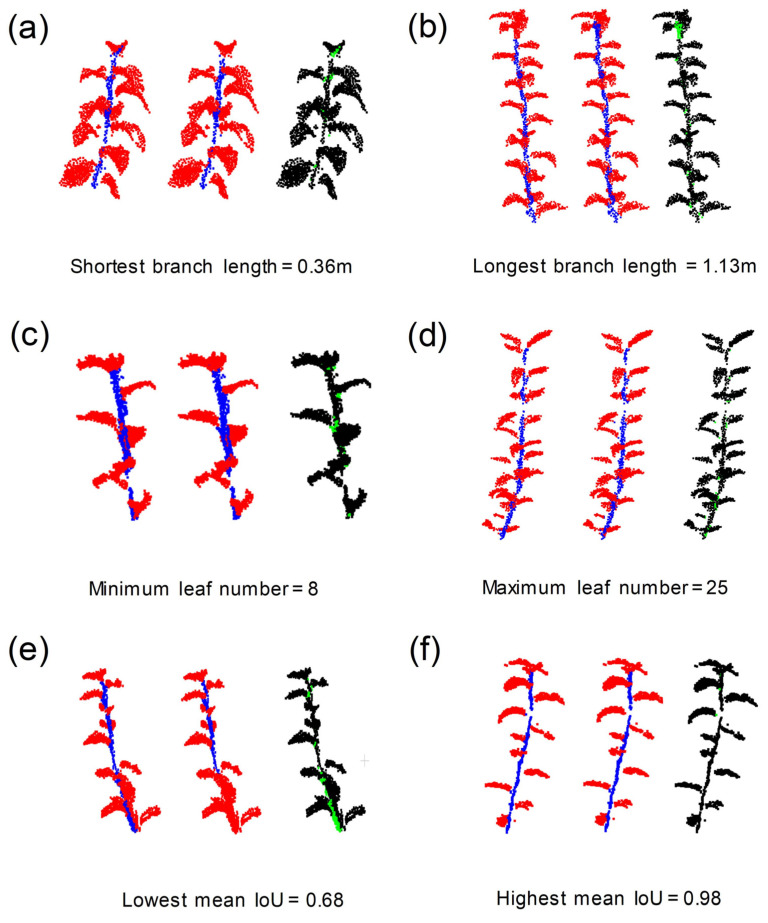
Visualization of branch–leaf semantic segmentation of branches with different attributes in the test dataset using PointNet++. In each subgraph, the left side shows the manual labeling, the middle shows the model prediction (branch and leaf points are in blue and red, respectively), and the right side shows the difference between them (same and different points of classification are in black and green, respectively).

The point clouds of 45 branch samples in the test dataset were segmented into branches and leaves, and the results were visually and quantitatively evaluated. Figure 5 shows the results of semantic segmentation of branches and leaves with different attributes, i.e., branch length and number of leaves, and the mean *IoU* of branch and leaf segmentation.

By observing the results of branch–leaf segmentation, we found that the output predicted by the model was very close to manual labeling. However, there were some subtle differences in the junction of branches and leaves, especially at regions near the top of the branch where the leaf clusters sheltered the branches. For instance, some leaves at the top of a branch (Figure 5b) were misclassified as branches, while the branches attached to the leaves (Figure 5e) were misclassified as leaves.

**Figure 6:**
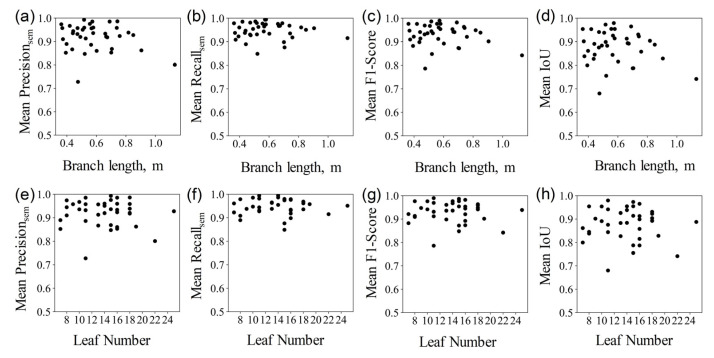
Distribution of the branch–leaf segmentation evaluation metrics with different branch lengths and numbers of leaves. Each subfigure shows mean *Precision*_sem_, mean *Recall*_sem_, mean *F1-score*, and mean *IoU* of each sample with different branch length (subfigures (**a**–**d**)) and leaf number (subfigures (**e**–**h**)), respectively.

The quantitative evaluation results are displayed in the Table 3. The mean *Precision*_sem_, mean *Recall*_sem_, mean *F*1*-score*, and mean *IoU* of the semantic segmentation of branches and leaves are 0.92 (Max: 0.99, Min: 0.73), 0.95 (Max: 0.99, Min: 0.85), 0.93 (Max: 0.99, Min: 0.79), and 0.88 (Max: 0.98, Min: 0.68), respectively. There are no significant differences in the results of infield scanning and indoor scanning for branch–leaf segmentation. Additionally, the segmentation results of this method for samples of different branch lengths and numbers of leaves showed little difference, demonstrating good robustness (as shown in Figure 6).

### 3.2. Single Leaf Segmentation

**Figure 7:**
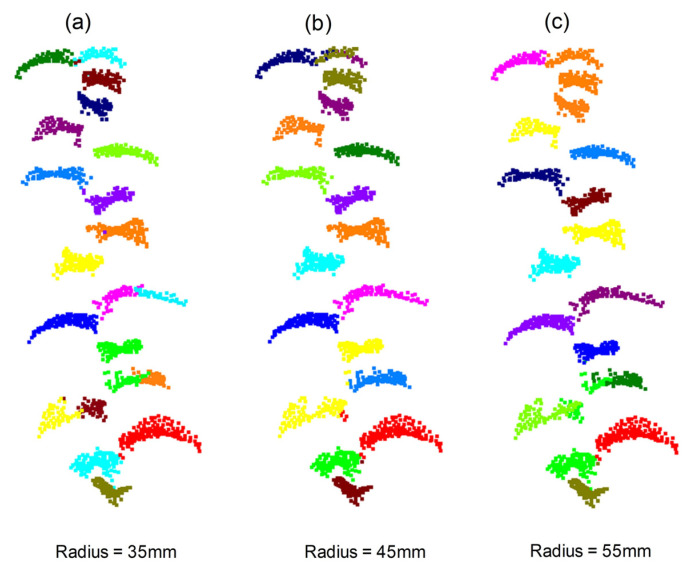
Examples of single leaf segmentation with different radius using mean shift clustering algorithms.

Based on the results of branch–leaf segmentation, we performed single leaf segmentation for leaf point clouds using mean shift clustering with different radii. From the examples presented in Figure 7, we found that after separating the branches and leaves in the previous step, most leaves could be segmented into single leaves through mean shift clustering. However, some small leaves with missing parts caused over-segmentation. When the radius was too small, one leaf was mistakenly divided into multiple leaves, as shown in Figure 7a. Conversely, when the radius was too large, two or more leaves were grouped into a single leaf. This problem was more obvious where there were leaves at the top of the branch, which were very small and close to each other, as shown in Figure 7c. We found that setting the radius to 45 mm (as shown in Figure 7b) achieved a more balanced segmentation effect.

**Figure 8:**
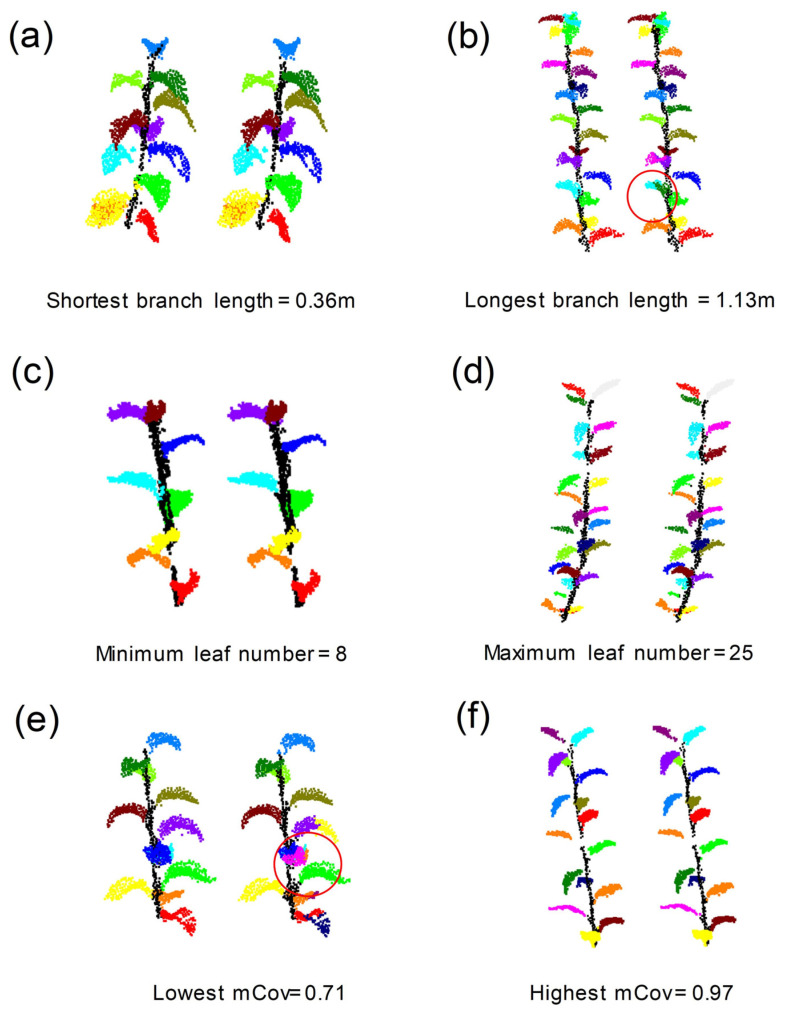
Visualization of single leaf segmentation of branches with different attributes using mean shift clustering algorithm (radius: 45 mm). In each subgraph, the left and right sides are the result of manual and automatic segmentation, respectively. Different leaves are represented by different colors.

To evaluate the segmentation effect, the instance-level precision (*Precision*_ins_), instance-level recall (*Recall*_ins_) and mean coverage (*mCov*) with different clustering radii were quantitatively evaluated. The segmentation results corresponding to different radii are shown in Table 4. The results show that, using the radius of 45 mm for mean shift clustering could better segment single leaves of pear tree branches. The single leaf segmentation of branches in the test dataset with different attributes (branch length and number of leaves) and *mCov* are shown in Figure 8. The results show that leaves can be separated from each other effectively with the given radius. However, some large leaves were mistakenly divided into two, as seen in Figure 8b,e.

**Figure 9:**
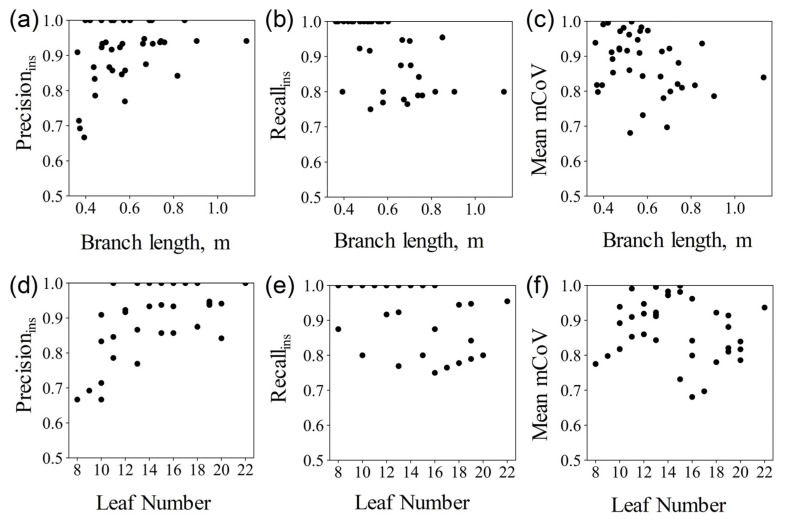
Distribution of the single leaf segmentation evaluation metrics with branch length and number of leaves. Each subfigure shows *Precision*_ins_, *Recall*_ins_, and mean *mCov* of each sample with different branch length (subfigures (**a**–**c**)) and leaf number (subfigures (**d**–**f**)), respectively.

The quantitative evaluation results presented in Table 5 show that the *Precision*_ins_, *Recall*_ins,_ and *mCov* of single leaf segmentation were 0.89 (Max:0.95, Min:0.68), 0.92 (Max:0.98, Min:0.74), and 0.87 (Max:0.97, Min:0.71), respectively. As well, the results of samples scanned indoors are better than infield for single leaf segmentation. For most samples, this method also showed good robustness of single leaf segmentation (as shown in Figure 9).

### 3.3. Esitimation of Phenotypic Parameters

**Figure 10:**
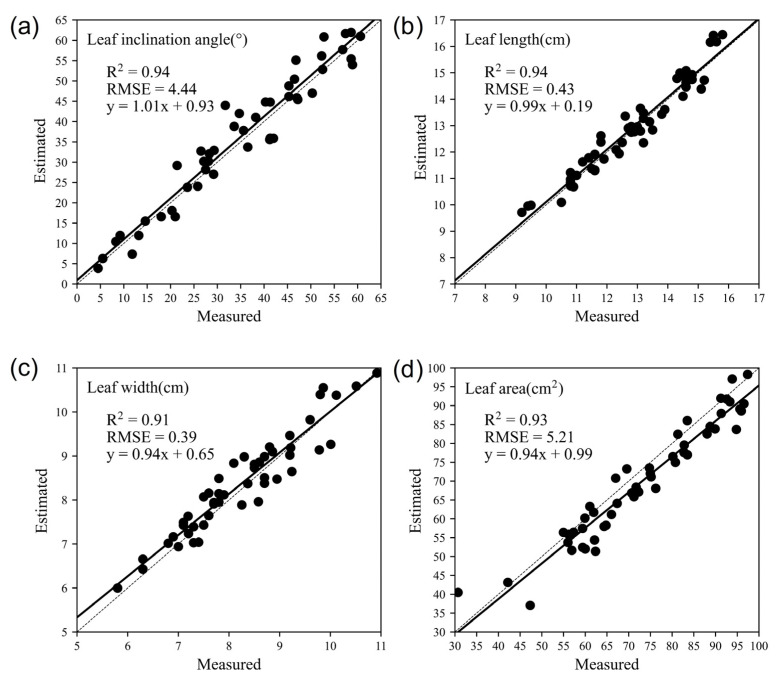
Comparison of phenotypic parameters estimated by the proposed method with the measured values: (**a**) leaf inclination angle; (**b**) leaf length; (**c**) leaf width; and (**d**) leaf area.

Based on the single leaf point cloud, the leaf inclination angle was calculated by plane fitting, and the leaf length, width, and area parameters were calculated by midrib fitting and triangulation. The estimated phenotypic parameters were compared with manual measurements to evaluate their accuracy by correlation analysis, as shown in Figure 10. The results show a high correlation between the estimated values and the measured values. For leaf inclination angle, the *R*^2^ and *RMSE* were 0.94 and 4.44°, respectively. The *R*^2^ and *RMSE* of leaf length were 0.94 and 0.43 cm, respectively, while the *R*^2^ and *RMSE* of leaf width were 0.91 and 0.39 cm, respectively. In addition, the *R*^2^ and *RMSE* of leaf area were 0.93 and 5.21 cm^2^, respectively. The midrib fitting method proposed in this study has obtained higher accuracy in estimating leaf length than in width, which may be due to leaf width estimation being more sensitive to curled leaves. In addition, the estimation of leaf area was slightly underestimated. In order to reduce the amount of calculation, we downsampled the original point cloud without changing its structure, which had no effect on the estimation of other phenotypic parameters except the leaf area. As shown in Figure 10d, the discrepancy between estimated and measured leaf area may be caused by the loss of boundary points of leaf point cloud after downsampling.

## 4. Discussion

### 4.1. Comparison of Similar Studies

The branch–leaf segmentation by PointNet++ achieved relatively high accuracy for pear trees. The mean *IoU* achieving in this study on branch–leaf segmentation reached 0.88, higher than that in literature [15], which used SegNet with Kinect V2 camera, and obtained mean *IoU* of 0.72. In addition to the processing method, terrestrial lidar is more effective to acquire the relatively complete and sophisticated three-dimensional canopy scans of (fruit) trees. However, the mean precision (0.95), recall (0.94), *F1-score* (0.95), and *IoU* (0.90) of branch (stem)–leaf segmentation by Psegnet [25] were slightly higher than our results (precision: 0.93, recall: 0.94, *F1-score* 0.93, and *IoU*: 0.88), owing to its special modules for plants, the double-neighborhood feature extraction block, the double-granularity feature fusion module, and the attention module. In the future, we will further improve accuracy by developing the appropriate modules for fruit trees.

Moreover, the single leaf segmentation by mean shift clustering also obtained acceptable results. In this study, the precision and recall of single leaf segmentation reached 0.89 and 0.92, higher than that in the literature [17], which obtained 0.78 and 0.87 (threshold = 8 mm) by using a geometric distance-based k–d tree. This is also slightly higher than that by Psegnet [25], which achieved highest precision of 0.90 and *mCov* of 0.85 (ours: 0.87) for tomato leaf, as well as highest recall of 0.82 for sorghum leaf. This may be because Psegnet is designed for segmenting point clouds of several different species of plants, while our approach is specifically for pear trees.

In terms of leaf phenotypic parameter estimation, the correlation coefficients of leaf length, leaf width, leaf inclination angle, and leaf area were 0.94, 0.91, 0.94, and 0.93, respectively. These are a little higher than the results for maize shoot in the literature [26], which were 0.90, 0.82, and 0.94 for leaf length, leaf width, and leaf inclination angle. Additionally, the correlation coefficients of leaf inclination angle and leaf area by our approach exceed those estimated in the literature [31], which were 0.90 and 0.87. Therefore, the phenotypic parameter estimation methods proposed in this study achieved high accuracy, especially for leaf length and width, using a midrib fitting algorithm.

### 4.2. Limitations

For single leaf segmentation, the proposed method performs better for indoor samples than infield. As shown in Table 3 and Table 5, for branch–leaf segmentation, the mean *F*1*-score* and mean *IoU* of infield and indoor samples were quite similar. At the same time, for single leaf segmentation, the indoor samples outperformed the infield samples for all the three metrics. In the branches’ dataset, point clouds in samples were occasionally incomplete due to the overlapping among branches and leaves. In general, due to no wind influence nor overlapping by other branches, the samples collected indoors were of higher quality than infield, mainly in the completeness and accuracy. The difference in data quality between infield and indoor samples mainly affected the single leaf segmentation, since branch and leaf segmentation used deep learning models which were trained with a large number of samples, including incomplete and non-smooth leaves. However, the single leaf segmentation used clustering, and the size of the incomplete leaves was smaller than the average size, so the clustering parameters were not appropriate for their segmentation, resulting in the deviation. In the future, we will integrate the single leaf segmentation with branch–leaf segmentation in the deep learning network to improve its robustness and expand its applications.

In addition, the leaf phenotypic estimation in this paper is more suitable for relatively complete leaves. In order to reduce the impact of branch and leaf occlusion on data acquisition, multi-angle imaging and multi-station lidar scanning are widely used in the construction of three-dimensional plant models. However, due to the serious overlap in fruit trees’ canopies, defects in point clouds occur even with multi-station lidar scanning. Incomplete leaves have a significant effect on estimation of phenotypic parameters. For example, if there are missing points in the base or tip of the leaf, the leaf length, width, and area estimated by the proposed midrib fitting algorithm will be underestimated. Currently, point cloud repair is the common approach to solve this problem, and our next plan is to repair the incomplete leaves to further improve the accuracy of estimation for leaf phenotypic parameters.

## 5. Conclusions

In this paper, we proposed an automatic pipeline for branch–leaf segmentation and leaf phenotypic parameter measurement for pear trees based on lidar point cloud. The method segments branch–leaf point clouds based on the PointNet++ model, extracts single leaf data by mean shift clustering algorithm, and estimates leaf inclination angle, length, width, and area by plane fitting, midrib fitting, and triangulation. It achieved high accuracy in branch–leaf segmentation, single leaf extraction, and leaf phenotypic parameter estimation. For branch–leaf segmentation, the mean *Precision*_sem_, *Recall*_sem_, *F*1*-score*, and *IoU* reached 0.93, 0.94, 0.93, and 0.88, respectively. For single leaf extraction, the *Precision*_ins_, *Recall*_ins_, and mean coverage (*mCov*) were 0.89, 0.92, and 0.87, respectively. The correlations between the estimated leaf inclination angle, length, width, and area and manual measurements were 0.94, 0.94, 0.91, and 0.93, respectively. The results demonstrate that the proposed pipeline could efficiently and accurately measure pear leaf phenotypic parameters, which could provide supporting data for monitoring pear tree growth, simulating canopy photosynthesis, and optimizing orchard management.

## Figures and Tables

**Table 1:** Specifications of lidar scanners used in this study.

Index	FARO Focus^3D^ S70
Laser Class	Level 1 (IEC 60825-1)
Laser wavelength	1550 nm
Detection range	0.6–70 m
Field of view	horizontal 360° × vertical 300°
Single point measuring speed	Up to 976,000 times/s
Scanner weight	4.2 kg
Sensors	Inclinometer, compass, GPS, height sensor, dual axis compensator
Scanning point spacing (scanning distance)	0.003 m (10 m)
Measuring error in distance	0.001 m

**Table 2:** Statistics of branch length and leaf number of samples in training set and test set.

Trait	Training Set				Test Set			
	Maximum Value	Minimum Value	Mean Value	Standard Deviation	Maximum Value	Minimum Value	Mean Value	Standard Deviation
Branch length (m)	1.06	0.25	0.57	0.16	1.13	0.36	0.58	0.17
Leaf number	26	8	14.6	3.6	25	8	14.3	3.7

**Table 3:** Evaluation of semantic segmentation of branches and leaves of samples in the test dataset.

Trait	Mean *Precision*_sem_			Mean *Recall*_sem_			Mean *F*1*-Score*			Mean *IoU*		
	Infield	Indoor	All	Infield	Indoor	All	Infield	Indoor	All	Infield	Indoor	All
Maximum	0.99	0.99	0.99	0.99	0.99	0.99	0.99	0.99	0.99	0.98	0.98	0.98
Minimum	0.46	0.85	0.73	0.58	0.78	0.85	0.62	0.81	0.79	0.45	0.71	0.68
Mean	0.92	0.94	0.92	0.95	0.93	0.95	0.93	0.93	0.93	0.88	0.89	0.88

**Table 4:** Evaluation of single leaf segmentation using mean shift clustering with different radii.

Radius/mm	*Precision*_ins_	*Recall*_ins_	*mCov*
35	0.79	0.74	0.76
45	0.89	0.92	0.87
55	0.73	0.82	0.72

**Table 5:** Evaluation of single leaf segmentation infield and indoors.

	*Precision*_ins_			*Recall*_ins_			*mCov*		
	Infield	Indoor	All	Infield	Indoor	All	Infield	Indoor	All
Maximum	0.96	0.98	0.95	0.98	0.99	0.98	0.96	0.98	0.97
Minimum	0.65	0.87	0.68	0.72	0.89	0.74	0.70	0.82	0.71
Mean	0.89	0.93	0.89	0.92	0.95	0.92	0.87	0.94	0.87

## Data Availability

The data presented in this study are available on request from the corresponding author.

## Abbreviations

The following abbreviations are used in this manuscript:

CC	Cloud Compare V2
PCL	Point Cloud Library
DBSCAN	Density-Based Spatial Clustering of Applications with Noise
KNN	K-Nearest Neighbor
SGD	Stochastic Gradient Descent
LS	Moving Least Squares
MLS	Least Squares
k–d tree	K-dimensional Tree
IoU	Intersection over Union
mCov	Mean Coverage
RMSE	Root Mean Square Error
